# Percutaneous Management of Cabrol Graft Chronic Total Occlusion: Case Report

**DOI:** 10.1016/j.jscai.2025.103824

**Published:** 2025-07-31

**Authors:** Teodora Donisan, Conor M. Lane, Christopher J. Francois, Guy Reeder, Malakh Shrestha, Rajiv Gulati, Abhiram Prasad

**Affiliations:** a Department of Cardiovascular Medicine, Mayo Clinic, Rochester, Minnesota; b Department of Radiology, Mayo Clinic, Rochester, Minnesota; c Department of Cardiovascular Surgery, Mayo Clinic, Rochester, Minnesota

**Keywords:** cabrol graft, case report, chronic total occlusion, multimodality imaging, percutaneous coronary intervention

## Abstract

Cabrol Dacron grafts are used to reimplant coronary arteries during aortic root replacement for complex aortic pathologies. Graft complications, particularly chronic total occlusions (CTO), are challenging. We present a 54-year-old man with a right coronary artery Cabrol graft CTO, treated successfully with percutaneous coronary intervention. Preprocedural imaging confirmed graft stump and right coronary artery collateral anatomy. Using advanced CTO techniques, the graft was recanalized, and symptoms improved. This is the first report of a Cabrol graft CTO percutaneous coronary intervention, demonstrating its feasibility and highlighting the importance of adjunctive imaging and need for hybrid antegrade CTO techniques.

## Introduction

The Cabrol procedure, introduced in 1981, uses a prosthetic graft for coronary reimplantation during aortic root replacement.^1^ This technique is employed in complex aortic pathologies and is associated with long-term graft patency issues, particularly in the right coronary artery (RCA).^2^ Although percutaneous coronary intervention (PCI) has been reported for Cabrol graft stenoses,^3^ occlusions have typically been managed surgically.^4^ We report the first successful PCI for an RCA Cabrol graft chronic total occlusion (CTO).

## Case presentation

A 54-year-old man with Canadian Cardiovascular Society class II angina, on metoprolol succinate 50 mg daily, was referred for evaluation. His history included 3 sternotomies: at age 22 years (*Streptococcus viridans* aortic valve endocarditis; he underwent homograft aortic valve replacement), at age 35 (severe aortic valve regurgitation treated with aortic valve replacement, 21-mm St. Jude mechanical valve [ Abbott]), and at age 52 (*Enterococcus faecalis* endocarditis treated with a Bentall procedure, 21-mm Konect conduit with a Resilia bioprosthetic valve [Edwards Lifesciences]). During his last surgery, the RCA was difficult to mobilize, and an 8-mm Gelweave Cabrol graft (Terumo) was utilized. One year postoperatively, he underwent coronary computed tomography angiography (CCTA) for exertional chest pain and was diagnosed with a periannular ring pseudoaneurysm and proximal RCA occlusion. Angiography confirmed the RCA CTO, and he was offered a fourth sternotomy with RCA bypass grafting but sought a second opinion.

Resting electrocardiography showed inferior T-wave inversions. Echocardiography revealed a left ventricular ejection fraction of 53% with septal and apical hypokinesis and akinesis. Positron emission tomography myocardial perfusion imaging showed a medium-sized reversible defect in the RCA territory, with reduced myocardial blood flow reserve (1.9). Cardiopulmonary exercise stress testing demonstrated reduced exercise capacity (38.7% of predicted, 3.8 metabolic equivalents of task), with a blood pressure decrease during peak exercise. After a heart team discussion, considering ischemia, angina burden, inability to escalate antianginal therapy (due to low baseline blood pressures and orthostatic symptoms), and surgical risk, PCI was pursued.

## Intervention

**Figure 1:**
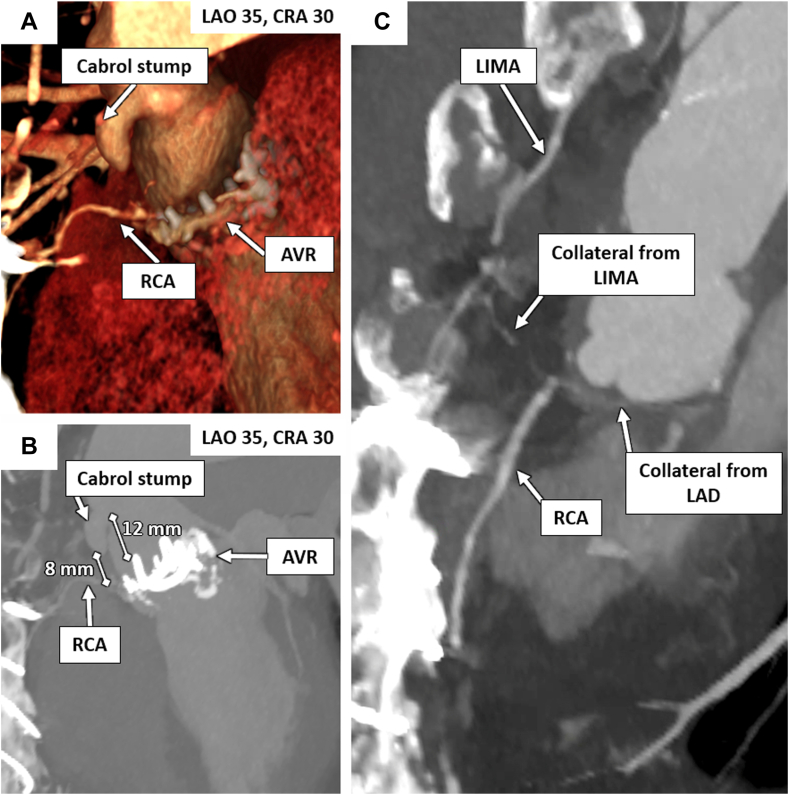
**Coronary computed tomography reconstruction demonstrating the Cabrol graft stump, the distance to the reconstituted RCA, the left to right collaterals, and the optimal angles for visualization**. AVR, aortic valve ring; CRA, cranial; LAD, left anterior descending coronary artery; LAO, left anterior oblique; LIMA, left internal mammary artery; RCA, right coronary artery.

Preprocedural CCTA showed reconstructions of the Cabrol stump (definitive CTO length 8 mm) and the collateral circulation to the proximal RCA (predominantly from the left anterior descending coronary artery; Figure 1). There was no radiographic visibility of the graft trajectory or the location of the RCA anastomosis (which could not be identified on the preprocedural CCTA either).

**Figure 2:**
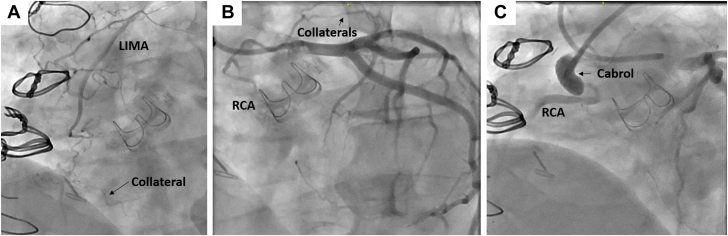
**Bilateral angiograms demonstratingthat the right coronary artery (RCA) filled via the collaterals just distal to the anastomosis of the Cabrol Gelweave graft**. We performed selective left internal mammary artery (LIMA, **A**) and left system injections, revealing that the dominant collateral is originating from the left anterior descending artery (**B**). The Cabrol CTO stump and an ambiguous path of the occlusion were defined (**C**).

**Figure 3:**
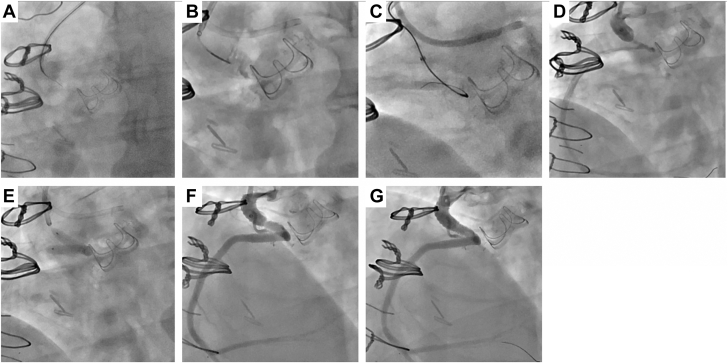
**Initial puncture of the proximal cap using Confianza Pro12 and advancement of a Turnpike Spiral microcatheter into the occlusion, hydrodynamic contrast injection performed for plaque modification** (**A, B**). Successful entry into the distal cap with the Confianza Pro12, crossing achieved with a Caravel microcatheter, and de-escalation to a Runthrough wire (**C, D**). Balloon dilation followed by deployment of a 4.0 × 38 mm Onyx stent with postdilation (**E**). Angiographic result demonstrating Thrombolysis in Myocardial Infarction (TIMI) 3 flow but proximal stent recoil (**F**). Placement of a second 5 × 8 mm Megatron stent, achieving final expansion and optimal result (**G**).

Left radial and right common femoral arteries were used for access (7F Slender and 7F sheaths, respectively) and bilateral angiograms were performed, showing small collaterals, unsuitable for retrograde techniques (Figure 2A-C; Supplemental Video 1). We noted a tapered proximal occlusion in the lower half of the Cabrol graft with blunt distal cap at the site of anastomosis with the RCA. As such, antegrade wire escalation was chosen as upfront crossing strategy. The Cabrol graft was engaged with a 7F multipurpose guide. Proximal cap penetration by 2 mm was successful using a Confianza Pro (CP12) wire (Asahi Intecc). Gladius, Gaia Third (both Asahi Intecc), and hydrodynamic contrast recanalization (0.5 mL of contrast injected; Supplemental Video 2) were used to advance through the occlusion. Due to the ambiguous path, a Gladius Mongo knuckle (Asahi Intecc) was advanced, but it did not enter the true lumen (Figure 3A, B). Penetration of the distal cap was successful with a CP12 (confirmed by retrograde angiography), crossed with a Caravel microcatheter (Asahi Intecc), and de-escalated to a Runthrough wire (Terumo) (Figure 3C, D). We noted significant angulation at the distal anastomosis site of approximately 90°. Serial predilation (2.5, 3.0, and 3.5 mm noncompliant balloons) restored Thrombolysis in Myocardial Infarction (TIMI) 2 flow. Significant lesion rigidity, recoil, and angulation necessitated a 7F TrapLiner (Teleflex) over an anchoring balloon for stent delivery. A 4.0 × 38 mm Onyx stent (Medtronic) was deployed at 22 atm and postdilated (4.0, 5.0 mm noncompliant balloons, 20 atm) (Figure 3E). Proximal stent recoil required an overlapping 5.0 × 8 mm Megatron stent (Boston Scientific) deployed at 20 atm, achieving full expansion and TIMI 3 flow (Figure 3F, G; Supplemental Videos 3 and 4). Intraprocedural proximal graft thrombus prompted tirofiban administration. The patient was discharged the following day on triple therapy (apixaban, aspirin, and clopidogrel), with aspirin discontinued after CYP2C19 genotyping demonstrated normal metabolized phenotype. One-month CCTA confirmed graft patency, without evidence of graft thrombus. Due to uncertainty about the initial mechanism of graft failure, presence of graft thrombus during the procedure, and patient preference, we decided to continue apixaban and clopidogrel for at least 6 months.

## Discussion

The Cabrol procedure has undergone multiple improvements to address reports of graft stenosis, thrombosis, and occlusion.^5^ Reoperations and inability to mobilize coronary buttons are common indications for a Cabrol graft. The 10-year incidence of coronary graft complications is 5.8% after a Cabrol procedure.^5^

RCA anastomosis is more susceptible to thrombosis or occlusion, likely due to blood flow alterations.^2^^,^^6^ Graft failure mechanisms include interposition graft or coronary ostium kinking, low coronary flow states due to small coronary ostia or excessive angulation, coronary ostium misalignment, arterial thrombosis, dissection, and excessive anastomosis tension with pseudoaneurysm.^7^ In our patient, the occlusion was identified 1 year after surgery. Potential mechanisms include graft thrombosis, accelerated atherosclerosis, and stenosis from kinking. These processes could have been triggered or enhanced by the significant angulation we noted at the distal anastomosis site, predisposing to nonlaminar flow. To address potential thrombosis, we pursued dual therapy (apixaban and clopidogrel). Although we did not use intracoronary imaging techniques in this case, these could have provided valuable insights into the composition of the lesion and its underlying mechanisms.

Cabrol graft occlusions are usually managed medically or surgically. This is the first case reporting successful CTO PCI for an occluded Cabrol graft. Preprocedural multimodality imaging was instrumental for assessing the graft, proximal RCA, and collaterals and for confirming viability and ischemia. Procedural risk was increased given the prior aortic surgeries, ambiguous graft path, noncoronary cusp nonthrombosed paravalvular pseudoaneurysm, and proximal RCA collaterals. The patient was counseled regarding risks, with the goal of relieving ischemia and angina.^8^^,^^9^ Given the lack of retrograde options, a hybrid antegrade approach was used, incorporating wire escalation, dissection, and the recently reported modified Carlino technique—hydrodynamic contrast recanalization.^10^ The latter technique involves synergistic use of contrast microinjections and polymer jacketed wires. Another notable aspect was the acute focal stent recoil within the graft, requiring a second layer of the high radial strength Synergy Megatron stent. Despite significant lesion complexity, an excellent procedural result was achieved.

## Conclusions

This case highlights the feasibility of PCI for Cabrol graft CTO through preprocedural planning, advanced imaging, and complex CTO techniques. A tailored, multidisciplinary approach is essential in managing challenging complications after Cabrol procedures. Our case adds to the growing body of evidence supporting PCI as a viable alternative in select, high-risk cases of prosthetic graft occlusions.
